# How should we give vitamin D supplementation? evaluation of the pediatricians’ knowledge in Turkey

**DOI:** 10.1186/s13052-017-0415-3

**Published:** 2017-10-17

**Authors:** Gizem Kara Elitok, Lida Bulbul, Umut Zubarioglu, Evrim Kıray Bas, Duygu Acar, Sinan Uslu, Ali Bulbul

**Affiliations:** 1 Department of Pediatrics, Sisli Hamidiye Etfal Education and Research Hospital, University of Health Sciences, Istanbul, Turkey; 2 Department of Pediatrics, Bakırköy Dr. Sadi Konuk Education and Research Hospital, University of Health Sciences, Istanbul, Turkey

**Keywords:** Child, Knowledge, Pediatrician, Vitamin D

## Abstract

**Background:**

We aimed to determine the knowledge and attitudes of Turkish pediatricians concerning vitamin D supplement.

**Methods:**

The study was planned cross-sectional to be carried out between April–May 2015 in Turkey. A questionnaire form that determined the participants’ opinions and practices concerning vitamin D supplement was completed via face-to-face interview.

**Results:**

A total of 107 pediatricians (49.3%) and 110 pediatric residents (50.7%) participated in the study. Of the physicians, 85.2% recommended vitamin D supplement for all infants and children regardless of diet, 13.4% recommended for the infants which are solely breastfed. Vitamin D supplement is recommended at a dose of 400 IU/day by 88.8% of pediatricians and by 90% of pediatric residents. Of the pediatricians and pediatric residents, 72% and 68.2%, respectively commence vitamin D supplement when the newborn is 15 days old. The rates of recommending vitamin D supplement until the age of one and two years were higher among pediatricians (48.6% and 41.1%, respectively) than pediatric residents (40.9% and 32.7%, respectively). The rate of starting vitamin D supplement for fontanelle closure was significantly higher among pediatric residents (15.5%) than pediatricians (3.7%) (*p* = 0.002). It was determined that the rate of prescribing vitamin D supplement until fontanelle closure was higher among pediatric residents (18.2%) than pediatricians (0.9%).

**Conclusions:**

The present study suggest that the knowledge of pediatricians about recommendation of vitamin D needs to be enhanced by education programs in addition to free vitamin D supplement provided by the Ministry of Health.

## Background

Vitamin D is a fat-soluble vitamin with hormone-like functions and plays an important role in bone-mineral metabolism. Detection of vitamin D receptors in many tissues of the body has produced new hypotheses on the functions of this vitamin. Today, discussion on this subject remains up-to-date because of documented relations between vitamin D deficiency and cancer, heart diseases, hypertension, diabetes, immune deficiency, chronic fatigue, obesity and autoimmune disorders [1, 2].

Serum 25-Hydroxy (OH) vitamin D level of an infant correlates with that of the mother in the first two months of life, whereas sunlight is more determinative in the following months [3, 4]. One liter of breast milk contains 12–60 IU vitamin D, which is not enough to meet daily requirement of vitamin D in infants. Likewise, the vitamin D contents of the foods are also inadequate. Therefore, vitamin D synthesis via sunlight and external vitamin D supplement gain importance during infancy [4–6].

Vitamin D deficiency and rickets remains to be a current problem in the developing countries as well as in the North American and European countries [7, 8]. In Turkey, the “Prevention of Vitamin D Deficiency and Protection of Bone Health Project” has been carried out since 2005 and, along with this project, 400 IU vitamin D supplement is provided free for all the infants until the age of 1 year (9). However, success of a vitamin D project in a country depends not only on the national consensus reports, but also on the attitudes of pediatric healthcare providers.

The present study aimed to determine the pediatricians’, who establish an important step in implementing and maintaining vitamin D supplement, knowledge and attitudes concerning vitamin D supplement in infants and children.

## Methods

The study was planned cross-sectional to be carried out between April and May 2015 in Turkey. The study sample consisted of 217 pediatricians, who agreed voluntarily to complete the questionnaire. Approval (490/2015) of the local ethics committee of Şişli Hamidiye Etfal Education and Research Hospital was obtained to conduct the study.

The questionnaire form was prepared by two pediatricians, who are experts on this subject, based on the recent literature. All participants were asked to complete a questionnaire form consisting of 13 questions via face-to-face interview. The questionnaire form includes the questions about academic title, the institution and the date of university graduate, as well as to which infants and children they recommend vitamin D supplement, why they recommend vitamin D supplement, when they recommend vitamin D supplement, for how long they recommend vitamin D supplement, whether they consider seasonal differences while recommending vitamin D supplement, by which method and at what dose they recommend vitamin D supplement.

### Study population

Pediatricians and pediatric residents in Turkey, who have been working actively were enrolled. The groups were compared in terms of the answers.

### Statistics

SPSS 15.0 for Windows program was used for the statistical analysis. Descriptive statistics were presented as a number and percentage for categorical variables. Comparison of ratios between two independent groups was calculated by Chi-square analysis. Monte Carlo simulation was used in the event conditions were not met. The statistical alpha level of significance was predetermined as *p* < 0.05.

## Results

A total of 264 subjects were interviewed over the course of study period. Thirteen subjects refused to participate and 11 subjects did not complete the questionnaire because of personal reasons. Of the 217 physicians who completed the study, 107 (49.3%) were pediatricians and 110 (50.7%) were pediatric residents. The demographic characteristics of the participants are shown in Table 1.

**Table 1:** Distribution of demographic characteristics of participants

		Number	Percent
Academic Title	Pediatric Resident	110	50.7
	Pediatrician	107	49.3
Institution	State Hospital	26	12
	Training and Research Hospital	152	70
	University Hospital	29	13.4
	Private Hospital	10	4.6
Time from university graduation	<5 Years	98	45.2
	5–10 Years	47	21.7
	11–20 Years	58	26.7
	>20 Years	14	6.5

The question “To which infants and children do you recommend vitamin D supplement?” was responded as “to all infants and children regardless of the type of diet” by 185 (85.2%), “to the infants receiving breast milk only” by 29 (13.4%), “to the infants receiving formula only” by 3 (1.4%) of all participants. The answers to the question “Why do you recommend vitamin D supplement to infants and children?” are demonstrated in Table 2.

**Table 2:** Distribution of the participants’ opinion on recommending vitamin D supplement

			Pediatric Resident	Pediatrician	p
			n(%)		
To which infants and children do you recommend vitamin D supplement?	Feeding	With breast milk only	16 (14.5)	13 (12.1)	
		Regardless of feeding	92 (83.6)	93 (86.9)	0.761
		With formula only	2 (1.8)	1 (0.9)	
	To the infants and children with inadequate exposure to the sunlight		26 (23.6)	25 (23.4)	0.127
	With disease likely to impair vitamin D metabolism		22 (20.0)	22 (20.6)	0.133
	Those that experience frequent respiratory tract infection		7 (6.4)	7 (6.5)	0.216
	Those with weakness and muscle pain		7 (6.4)	8 (7.5)	0.200
Why do you recommend vitamin D supplement to the infants and children?	To prevent rickets and vitamin D deficiency		97 (88.2)	104 (97.2)	0.008
	For fontanelle closure		17 (15.5)	4 (3.7)	0.002
	To prevent infections		14 (12.7)	26 (24.3)	0.013
	Other		2 (1.8)	9 (8.4)	

Other: Because breast milk contains inadequate vitamin D (*n* = 3), for protection against allergic diseases (n = 2), to prevent depression (n = 2), because the Ministry of Health supplies vitamin D for free (*n* = 2), to support the immune system (n = 1), to enable earlywalking (*n* = 1)

The rate of prescribing vitamin D preparation for fontanel closure was significantly higher among pediatric residents (15.5%) as compared to the pediatricians (3.7%) (*p* = 0.002). However, it was determined that prescribing vitamin D preparation to prevent rickets and vitamin D deficiency was more common among pediatricians (97.2%) than pediatric residents (88.2%) (*p* = 0.008). The rate of recommending vitamin D supplement for the prevention of infections was higher among pediatricians (24.3%) as compared to the pediatric residents (12.7%) (*p* = 0.013).

Distribution of the responses given to the questions about the time, duration, method and dose of vitamin D supplement, as well as the seasonal differences in the dose of vitamin D supplement is demonstrated in Table 3. There was statistically significant difference between the groups in terms of recommended duration of vitamin D supplement (*p* = 0.001). The rate of recommending vitamin D supplement until 1 and 2 years of age was higher for pediatricians than pediatric residents (Table 3).

**Table 3:** Distribution of the participants’ opinions on the time, duration, method and seasonal differences of recommending vitamin D supplement

		Pediatric resident	Pediatrician	p
		n (%)		
When do you recommend to start vitamin D supplement?	At birth	24 (21.8)	26 (24.3)	0.186
	On the 15th day after birth	75 (68.2)	77 (72.0)	
	In a month after birth	5 (4.5)	4 (3.7)	
	When the infant is 3-month-old	5 (4.5)	0 (0.0)	
	Other	1 (0.9)	0 (0.0)	
For how long do you recommend vitamin D supplement?	Until the closure of fontanelle	20 (18.2)	1 (0.9)	0.001
	Until the 6th month of life	2 (1.8)	1 (0.9)	
	Until the age of 1 year	45 (40.9)	52 (48.6)	
	Until the age of 2 years	36 (32.7)	44 (41.1)	
	Until the age of 3 years and longer	7 (6.4)	9 (8.4)	
At what dose do you recommend vitamin D supplement?	Daily 200 IU	2 (1.8)	0 (0.0)	0.336
	Daily 400 IU	99 (90)	95 (88.8)	
	Daily 600 IU	6 (5.5)	8 (7.5)	
	Breaking an ampoule and receiving orally	1 (0.9)	0 (0.0)	
	No comment	2 (1.8)	4 (3.7)	
Do you recommend different dose of vitamin D depending on the season (summer-winter)?	Only in winter	7 (6.4)	0 (0.0)	0.012
	Entire year, higher in winter	45 (40.9)	37 (34.6)	
	Entire year at the same dose	58 (52.7)	70 (65.4)	
Method of vitamin D supplement?	Drop	92 (83.6)	90 (84.1)	0.521
	Drop + multivitamin	10 (9.1)	12 (11.2)	
	Drop + ampoul	2 (1.8)	0 (0.0)	
	Multivitamin	6 (5.5)	5 (4.7)	

Statistical difference was determined between the answers given to the question “Do you recommend different doses of vitamin D in winter and in summer?” (*p* = 0.012). The rate of prescribing the same dose of vitamin D all year long was higher among the pediatricians as compared to the pediatric residents (Table 3).

## Discussion

Even though Turkey has high levels of sun exposure, vitamin D deficiency remains to be an important problem for pregnant women, infants and adolescents. The studies reported that the prevalence of rickets in Turkish children younger than the age of 3 years-old ranges between 1.67% and 19% [9–11]. In a study conducted in 2002–2003 in Ankara, the prevalence of rickets was reported to be 6.8% [12]. The prevalence of rickets has shown a remarkable decrease when the Vitamin D supplement program was initiated in 2005 by the Ministry of Health [9]. Accordingly, Özkan et al. reported that the prevalence of rickets in Erzurum, which was 6% before the program, significantly decreased to 1.0% after the program [11, 13]. However, although the prevalence of rickets decreased, the prevalence of vitamin D deficiency remains high. In the trial carried out in 2011 by the Ministry of Health in collaboration with Gazi University (Ankara), vitamin D deficiency (threshold for 25 OH vitamin D deficiency: <15 ng/ml) was detected in 26.8% of the infants aged 6-to-17 months. According to the statements of the mothers in that study, only 67% of the infants had received vitamin D as supplement or treatment and, 48% have discontinued vitamin D supplement at the recommendation of physician [14]. Therefore, within the strategy of prevention of vitamin D deficiency, the present study not only provided free vitamin D supplement, but also revealed the necessity of health care providers’, especially pediatricians, to participate in education programs concerning continuation of vitamin D supplement. In Turkey, there are limited number of studies on the knowledge and attitudes of health care providers, particularly in the field of pediatrics, concerning vitamin D supplement. Herein, this study aimed to determine the knowledge and attitudes of pediatricians concerning vitamin D supplement via face-to-face interview.

The infants fed with formula needed to consume 1 l of formula in order to receive an adequate amount of vitamin D [15]. Therefore, recommending vitamin D supplement to the infants solely breastfed and not to those fed with formula/mixed (breast milk + formula) should not be considered as a proper practice in terms of vitamin D supplement. According to the earlier studies and consensus reports, vitamin D supplement is recommended for all infants after birth regardless of the type of diet [3, 9, 16]. Toprak et al. determined that 54% of the primary care physicians adhere to this consensus [17]. Pehlivan et al. determined that the majority of pediatricians (85%) behave in accordance with this consensus [18]. Similarly, this study found that the majority of pediatricians and pediatric residents behave in accordance with the consensus report. However, this study determined that a considerable proportion of the pediatricians and pediatric residents recommend vitamin D supplement to the infants fed with breast milk only.

Vitamin D deficiency can be seen in the chronic diseases of the tissues and organs and various medications can also lead to vitamin D deficiency. Antiepileptics, steroids and chemotherapeutic drugs are known to have unfavorable effects on bone development and formation [15, 19]. In this respect, vitamin D supplement needs to be provided for those having any disease likely to impair vitamin D metabolism (celiac disease, cystic fibrosis, chronic liver diseases, etc.). It should be kept in mind that these children may still develop vitamin D deficiency despite 400 IU/day vitamin D supplement. In such children, the dosage of vitamin D supplement must be increased [15, 20]. In this study, the rate of recommending vitamin D supplement to the “children with any disease likely to impair vitamin D metabolism” was low among both pediatricians and pediatric residents.

There are various suggestions on the dose of vitamin D supplement. In a comparative analysis of nutritional guidelines worldwide in 2017, the average recommended dose of vitamin D is 400 IU per day [20]. The National Academy of Medicine (Institute of Medicine) recommends vitamin D supplement at a dose of 400 IU/day before the age of one year and 600 IU/day after the age of one year [21]. Likewise, Turkish Pediatric Endocrinology Society consensus report recommends vitamin D supplement at a dose of 400 IU/day [9]. This study determined that the majority of pediatric residents and pediatricians recommend vitamin D supplement at a dose of 400 IU/day however, a very small proportion of pediatric residents recommend vitamin D supplement at a dose of 200 IU/day.

Maternal vitamin D deficiency was reported to be between 55% and 81% in Turkey [22–24]. A study from in Turkey demonstrated that maternal serum 25 OH vitamin D level < 10 ng/ml is the most significant risk factor for low level of neonatal 25 OH vitamin D (OR = 15.2, *p* = 0.02) [25]. For this reason vitamin D supplement is recommended from the first days of life [6, 9, 15]. Toprak et al. reported that 14% of primary care physicians prescribes vitamin D supplement from the first day of life, whereas 41% prescribe within the first month [17]. This study determined that vitamin D supplement is started on the 15th day after birth by substantial proportion of pediatricians and pediatric residents but the rate of starting vitamin D supplement on the first day of life is low.

There are different suggestions on the duration of vitamin D supplement. The American Academy of Pediatrics recommends vitamin D supplement from the first few days of life and through adolescence [15]. Toprak et al. determined that 39% of primary care physicians recommend vitamin D supplement for at least one year. The same study found that 21% of primary care physicians prescribes vitamin D supplement for 6 months, 7.4% prescribes for 9 months, and the rate of prescribing vitamin D supplement up to 36 months is just 1% [17]. This study determined that nearly half of the pediatricians and pediatric residents recommend vitamin D supplement until the age of one year but a small percentage recommend vitamin D supplement until the age of two years. From these results it appears that education programs are required in order to prolong the duration of vitamin D supplement in Turkey.

As brain development is faster in the first two years of life, regular monitoring of head circumference is important in the presence of suspicious situations [26]. The fontanelle might be larger than normal in rickets cases and delayed closure may be encountered [27, 28]. There is no study in the literature concerning the effect of vitamin D supplement on early fontanelle closure at maintenance dosage. Accordingly discontinuing vitamin D supplement because of small fontanelle, as well as giving vitamin D supplement until the closure of fontanelle is not a good practice. In this study, the rate of recommending vitamin D for “fontanelle closure” was statistically higher among pediatric residents as compared to the pediatricians. It was determined that substantial proportion of pediatric residents recommends vitamin D supplement “until fontanel closure”, which may lead to early discontinuation of vitamin D supplement in the infants with anterior fontanelle closure before the age of one year.

There are studies reporting that a 400 IU/ day vitamin D supplement remains inadequate because 25 OH vitamin D levels decrease in winter [29, 30]. For this reason the Canadian Association of Pediatrics recommend vitamin D supplement at a dose of 800 IU/ day between October and April, particularly for those living in the northern hemisphere (55th parallel northand over) [31]. Mutlu et al. demonstrated that regular vitamin D supplement given at a dose of 400 IU/day increases 25 OH vitamin D level over 20 ng/ml [32]. Observing a decrease in the incidence of rickets along with 400 IU/day vitamin D supplement program suggests that vitamin D received at a dose of 400 IU/day regardless of seasonal difference is effective in preventing vitamin D deficiency in Turkey [9]. This study determined that the majority of pediatricians and half of the pediatric residents recommend equal dose of vitamin D supplement regardless of seasonal difference in accordance with the consensus report. However, pediatric residents, even though at a low rate, recommend vitamin D supplement only in winter.

## Conclusion

This study revealed that the knowledge and attitudes of pediatricians and pediatric residents in Turkey concerning vitamin D supplement are not at targeted levels although they are in line with consensus reports and vitamin D supplement program initiated by Turkish Ministry of Health. Data of this study suggest that the knowledge of pediatricians (pediatric residents in particular) about application of vitamin D supplement, time for prescribing and duration of vitamin D supplement needs to be enhanced. With regard to the prevention of vitamin D deficiency, the present study set forth the necessity of education programs for health care providers in addition to free vitamin D supplement provided by Turkish Ministry of Health.

## Abbreviation

25 OH vitamin D: 25-hydroxy vitamin D
